# Utilization of health information technology among cancer genetic counselors

**DOI:** 10.1002/mgg3.1315

**Published:** 2020-05-28

**Authors:** Jordon B. Ritchie, Caitlin G. Allen, Heath Morrison, Michelle Nichols, Steven D. Lauzon, Joshua D. Schiffman, Chanita Hughes Halbert, Brandon M. Welch

**Affiliations:** ^1^ Department of Public Health Sciences Medical University of South Carolina Charleston SC USA; ^2^ Biomedical Informatics Center Medical University of South Carolina Charleston SC USA; ^3^ Department of Behavioral Sciences and Health Education Emory University Atlanta GA USA; ^4^ College of Nursing Medical University of South Carolina Charleston SC USA; ^5^ University of Utah Family Cancer Assessment Clinic Huntsman Cancer Institute Salt Lake City UT USA; ^6^ Department of Psychiatry and Behavioral Sciences Medical University of South Carolina Charleston SC USA; ^7^ Medical University of South Carolina Hollings Cancer Center Charleston SC USA

**Keywords:** biomedical informatics, genetic counseling, health information technology, software

## Abstract

**Background:**

Health information technology (IT) is becoming increasingly utilized by cancer genetic counselors (CGCs). We sought to understand the current engagement, satisfaction, and opportunities to adopt new health IT tools among CGCs.

**Methods:**

We conducted a mixed‐mode survey among 128 board‐certified CGCs using both closed‐ and open‐ended questions. We then evaluated the utilization and satisfaction among 10 types of health IT tools, including the following: cancer screening tool, family health history (FHx) collection tools, electronic health records (EHRs), telegenetics software, pedigree drawing software, genetic risk assessment tools, gene test panel ordering tools, electronic patient education tools, patient communication tools, and family communication tools.

**Results:**

Seven of 10 health IT tools were used by a minority of CGCs. The vast majority of respondents reported using EHRs (95.2%) and genetic risk assessment tools (88.6%). Genetic test panel ordering software had the highest satisfaction rate (very satisfied and satisfied) at 80.0%, followed by genetic risk assessment tools (77.1%). EHRs had the highest dissatisfaction rate among CGCs at 18.3%. Dissatisfaction with a health IT tool was associated with desire to change: EHRs (*p* < .001), cancer screening tools (*p* = .010), genetic risk assessment tools (*p* = .024), and family history collection tools (*p* = .026). We found that nearly half of CGCs were considering adopting or changing their FHx tool (49.2%), cancer screening tool (44.9%), and pedigree drawing tool (41.8%).

**Conclusion:**

Overall, CGCs reported high levels of satisfaction among commonly used health IT tools. Tools that enable the collection of FHx, cancer screening tools, and pedigree drawing software represent the greatest opportunities for research and development.

## Graphical abstract

The utilization and satisfaction of health IT tools among cancer genetic counselors varies. Health IT tools that enable the collection of family health history, cancer screening, pedigree management, and telegenetics represent the greatest opportunities for research and development.

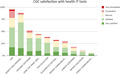

## 1 INTRODUCTION

Health information technology (IT) is used across healthcare for clinical documentation, to streamline clinical workflows, improve quality of care, increase patient safety, facilitate communication, and support clinical decision‐making (Wager, Lee, & Glaser, 2017). For over two decades, health IT has been available to cancer genetic counselors (CGCs) for use across the CGC workflow (Gordon, Babu, & Laney, 2018; Welch & Kawamoto, 2012). Health IT can be used prior to a genetic counseling appointment to identify high‐risk patients and to collect personal and family history information from patients (Welch et al., 2018). Health IT can also be used during counseling to document the patient visit, calculate disease risk, order genetic tests, and meet with patients remotely (Aronson et al., 2016). In addition, health IT can be used after the counseling visit to coordinate care with other providers, communicate genetic test results with patients, and help patients share test results with their relatives (Lynch et al., 2014).

Given the ability of health IT to influence many aspects of genetic counseling for cancer, it is necessary to understand and characterize the utilization of health IT by CGCs. While some studies have explored utilization of specific technologies, (Goehringer et al., 2018; Welch et al., 2018; Zuniga, 2018) none have taken a comprehensive health IT perspective. The objectives of our research study were to: (1) understand how commonly various health IT tools are being used in the day‐to‐day practice of CGCs, (2) assess CGCs’ satisfaction with these tools, and (3) assess CGCs’ interest in adopting new health IT tools or change the health IT they use. This information can be used to inform decisions about whether CGCs are likely to adopt certain health IT tools and where health IT tool research and development are most needed.

## 2 METHODS

We conducted a mixed‐mode survey, including both closed‐ and open‐ended questions, among English‐speaking, board‐certified CGCs who specialize in providing cancer care to patients (defined as at least 50% of their patient load) in a U.S. healthcare organizations (Dillman, Smyth, & Christian, 2008). CGCs who work for genetic testing companies or those not actively seeing patients in a clinical setting (e.g., retired, maternity leave) were not included in the study. Data were collected via mixed‐modes through in‐person and electronic data capture procedures. Initially, study participants were recruited at the 2018 National Society of Genetic Counselors conference in Atlanta, GA. Enrollees completed surveys in‐person facilitated by a study team member who asked the questions and entered data into REDCap (version 8.6.5) (Harris et al., 2009). Next, a recruitment email with a link to the REDCap survey was distributed to 757 genetic counselors listed on the National Cancer Institute Cancer Genetics Services Directory (“NCI Cancer Genetics Services Directory”, 2011). The respondents recruited via email had access to the same questions as those who completed the survey in‐person, although their responses were entered without assistance. We used the mixed‐mode approach to verify consistency of data, while minimizing overall study time costs from a broader group. Study participants were compensated with a $10 gift card. The study protocol was reviewed and approved as an exempt study by the Institutional Review Board at the Medical University of South Carolina (MUSC). All participants gave their informed consent prior to their inclusion in the study.

### 2.1 Health IT tools assessed

After consulting relevant literature on health IT tools in genetic counseling, we identified 10 unique health IT tools that could be used during the provision of genetic counseling: cancer screening tools, (Hampel, Sweet, Westman, Offit, & Eng, 2004) family health history (FHx) collection tools, (Welch et al., 2018) electronic health records (EHRs), (Belmont & McGuire, 2009) telegenetics software, (Hilgart, Hayward, Coles, & Iredale, 2012; Zuniga, 2018) pedigree drawing software, (Welch et al., 2018) genetic risk assessment tools, (Antoniou, Pharoah, Smith, & Easton, 2004; Chipman et al., 2013; Tyrer, Duffy, & Cuzick, 2004) gene test panel ordering tools, (Aronson et al., 2016) patient education tools, (Green, McInerney, Biesecker, & Fost, 2001) patient communication tools, (Lynch et al., 2014), and family communication tools (Hughes et al., 2002). See Table 1 for descriptions of the clinical purpose of each type of health IT tool.

**TABLE 1:** Health IT categories and clinical purposes used in the survey questions

Health IT tool	Clinical purpose
Cancer screening	Identify high‐risk patients among the general cancer population
FHx collection	Collect family health history from patients
EHR	Document patient visits
Telegenetics software	Meet with patients remotely
Pedigree drawing software	Assist pedigree drawing, documentation, and management
Genetic risk assessment	Calculate hereditary cancer or gene carrier risk
Gene test panel ordering	Facilitate gene test/panel ordering, paperwork, and consent
Patient education	Deliver educational resources to patients
Patient communication	Facilitate patient communication and/or disclosure of test results
Family communication	Help patients share genetic test results with family members

Abbreviations: EHR, electronic health record; FHx, family health history.

### 2.2 Assessment

For each health IT tool, we asked the same series of questions, modifying the questions according to the specific health IT tool assessed. For each tool, we asked, “Do you/your organization currently use {health IT tool} to {clinical process}?” Available answers were “yes” and “no.” If the respondent answered yes, we asked “What {health IT tool} do you use to {clinical purpose}?” Subsequently, participants were asked about their level of satisfaction with the tool: “How satisfied are you with {response above} to {clinical purpose}?” Response selections used a 5‐point Likert scale with options ranging from “very dissatisfied” to “very satisfied.” These questions were followed by two open‐ended questions: “What do you like about it?” and “What can be improved?” We then asked whether they were considering switching to another health IT tool for that specific clinical purpose, allowing either yes/no responses. For those answering affirmatively, we asked additional open‐ended questions to identify what other tools they were considering and why they thought those tools were better. We also asked them what barriers, if any, would prevent them from switching to what they considered to be a better tool.

If a respondent answered “no” to the first question (“Do you/your organization currently use {health IT tool} to {clinical process}?”), we asked two open‐ended questions: “Why not?” and “How do you currently {clinical purpose}?” Next we asked “Are you considering using a {health IT tool} to {clinical purpose}?” with two options “yes, currently considering it” and “no, not currently considering it.” If the respondent responded “yes” to this option, we asked three open‐ended questions: “What products are you considering? Why?” and “What barriers, if any, prevent you from adopting it?” If they answered “no” to the previous question, we asked “Why not?” Open‐ended survey responses will be qualitatively coded and reported elsewhere.

### 2.3 Data analysis

Descriptive statistics are presented as *n* (%) for categorical variables. Chi‐squared and Fisher's exact test were used for categorical variables, as appropriate. To determine the influence of the Likert‐based satisfaction score for each health IT tool on whether or not the respondents would consider switching to another health IT tool, regressions were performed in order to determine whether differences observed reached the threshold of statistical significance. All regression models were verified to meet standard modeling assumptions. Mean differences in responses as well as confidence intervals for mean differences in response among those who would and would not consider switching to another health IT Tool as well as p‐values are given for each health IT tool. Statistical significance was assessed with an α‐level of 0.05. Statistical analysis was performed using R software (version 3.4.3, R Core Team) (R Core Team, 2013).

## 3 RESULTS

A total of 142 genetic counselors consented to participate in the survey, of which 140 completed demographic information. Between 121 and 128 answered questions related to health IT tools. Just over half (55.7%) of respondents reported working at a public or private hospital or medical facility where they provided patient care directly to patients, 53 (37.9%) worked at a university medical center, and nine (6.4%) worked for a private genetic counseling company. Overall, respondents reported working an average of 8.1 years as a CGC and have worked at their current organization an average of 6.1 years.

### 3.1 Utilization of health IT

**FIGURE 1:**
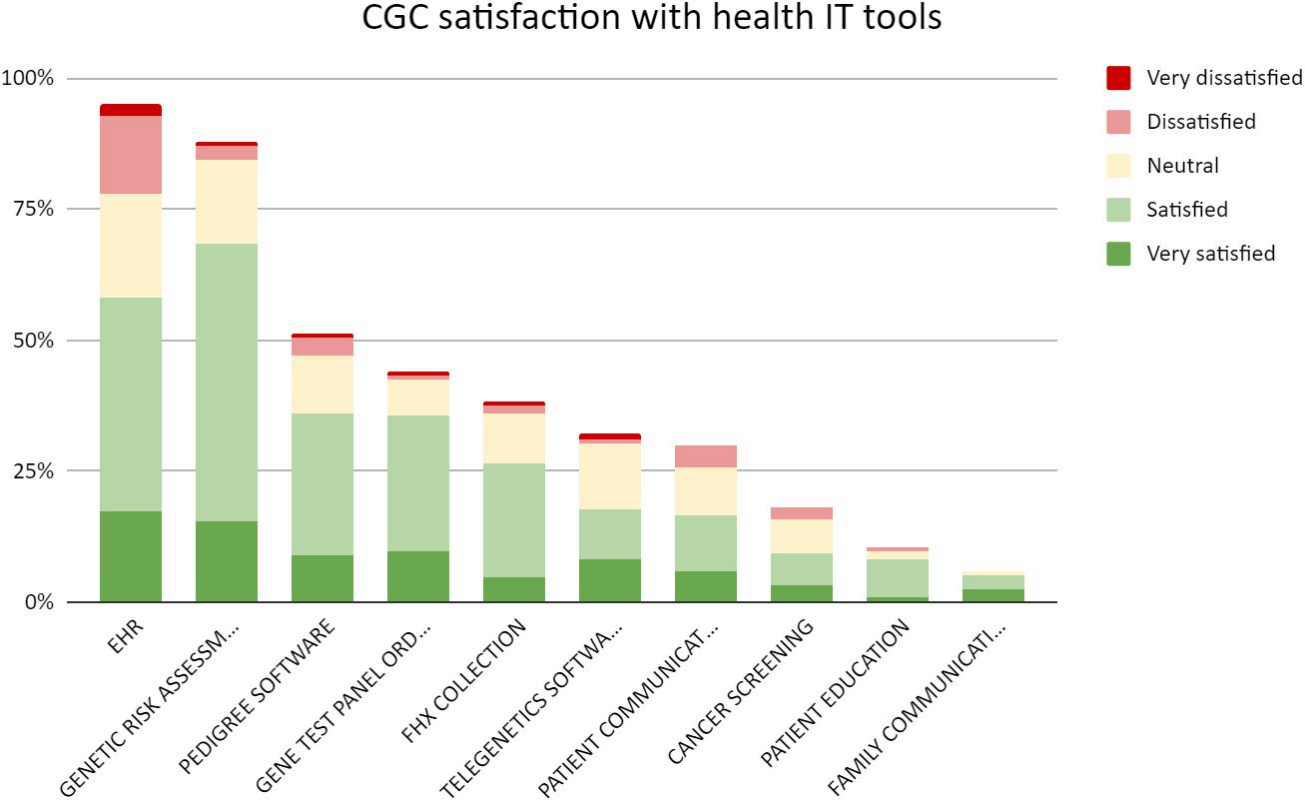
CGC Satisfaction with health IT tools

The utilization of health IT tools was low overall (Table 2). Seven of 10 health IT tools we assessed were used by only a minority (less than 50%) of CGCs currently (Figure 1). EHRs were the most widely used (95.2%) health IT tool by CGCs and also had the highest variety (*n* = 19) of EHR products. Despite this variety, nearly two thirds (61.1%) used Epic™. About 88.6% of CGCs reported using a health IT tool to calculate hereditary cancer risk. Respondents reported using 12 different cancer or genetic risk assessment tools, with most (88.1%) users using International Breast Cancer Intervention Study (IBIS) breast cancer risk assessment tool (i.e, Tyrer‐Cuzick model) (Tyrer et al., 2004). Just over half (52.8%) of CGCs reported using pedigree drawing software, 69.7% of which used Progeny™. Less than half (44.7%) used a health IT tool to facilitate gene test panel ordering, with most (84.9%) using a laboratory's online portal. Only 39.1% of CGCs used a FHx collection tool, the majority of whom (55.3%) use Progeny™ Family History Questionnaire. One third (33.6%) of CGCs reported using telegenetics software, with one third (29.3%) of users unsure which telehealth software they use. About one third (30.6%) of CGCs used health IT to facilitate communication with a patient, with the majority (73.0%) using patient communication capabilities within their EHR. Few CGCs used a cancer screening tool (18.0%), patient education tool (10.6%), and family communication tool (5.8%).

**TABLE 2:** Rates of users, level of satisfaction, and adoption among participants

Health IT tool	Users	Satisfied	Dissatisfied	Users seeking to change	Likely to change due to dissatisfaction (p‐value)	Nonusers seeking to adopt	Total considering changing or adopting	Projected adoption
EHR	95.2% (120/126)	60.8% (73/120)	18.3% (22/120)	10.0% (12/120)	0.001	100.0% (6/6)	14.3% (18/126)	100.0% (126/126)
Genetic risk assessment	88.6% (109/123)	77.1% (84/109)	3.7% (4/109)	13.1% (14/107)	0.024	21.4% (3/14)	14.0% (17/121)	91.1% (112/123)
Pedigree drawing software	52.8% (66/125)	68.2% (45/66)	7.6% (5/66)	31.3% (20/64)	0.16	53.4% (31/58)	41.8% (51/122)	77.6% (97/125)
Gene test panel ordering	44.7% (55/123)	80.0% (44/55)	3.6% (2/55)	5.5% (3/55)	0.94	13.6% (9/66)	9.9% (12/121)	52.0% (64/123)
FHx collection	39.1% (50/128)	68.0% (34/50)	6.0% (3/50)	22.4% (11/49)	0.026	66.2% (51/77)	49.2% (62/126)	78.9% (101/128)
Telegenetics software	33.6% (42/125)	52.4% (22/42)	4.8% (2/42)	10.3% (4/39)	0.76	50.0% (41/82)	37.2% (45/121)	66.4% (83/125)
Patient communication	30.6% (37/121)	54.1% (20/37)	13.5% (5/37)	5.6% (2/36)	0.09	8.5% (7/82)	7.6% (9/118)	36.4% (44/121)
Cancer screening	18.0% (23/128)	52.2% (12/23)	13.0% (3/23)	21.7% (5/23)	0.010	50.0% (52/104)	44.9% (57/127)	58.6% (75/128)
Patient education	10.6% (13/123)	76.9% (10/13)	7.7% (1/13)	15.4% (2/13)	0.59	23.4% (25/107)	22.5% (27/120)	30.9% (38/123)
Family communication	5.8% (7/121)	85.7% (6/7)	0.0% (0/7)	0.0% (0/7)	—	14.8% (16/108)	13.9% (16/115)	19.0% (23/121)

Abbreviations: EHR, electronic health record; FHx, family health history.

### 3.2 Satisfaction with health IT

Overall, genetic counselors were more satisfied (satisfied and very satisfied) (52.2%–80%) with all health IT tools they used than dissatisfied (dissatisfied and very dissatisfied) (0%–18.3%). Among health IT tools used by at least a quarter of respondents, genetic test ordering software had the highest rate of satisfaction (80.0%), followed by genetic risk assessment tools (77.1%) among users. EHRs had the highest dissatisfaction rate (18.3%) among health IT tools, followed by patient communication tools (13.5%).

### 3.3 Genetic counselors seeking to change or adopt

One third (31.3%) of pedigree drawing software users and 22.4% of FHx collection tool users were considering changing or switching to another solution (Table 2). Only 5.5% of gene test panel ordering tool users were seeking to change. Despite having the second‐highest dissatisfaction rate, only 5.6% of those that used patient communication tools were seeking to change. Dissatisfaction with a health IT tool was associated with a statistically significant (*p* < .05) desire to change for EHR (*p* = .001), cancer screening (*p* = .010), genetic risk assessment (*p* = .024), and FHx collection tools (*p* = .026).

All nonusers of EHRs (100%) and a majority of nonusers of FHx collection tool (66.2%), pedigree drawing software (53.4%), cancer screening tools (50%), and telegenetics software (50%) were seeking to adopt the technology. Only a relatively small portion of nonusers of patient communications tools (8.5%), gene test panel ordering tools (13.6%), and family communication tools (14.8%) were seeking to adopt a health IT tool.

About 49.2% of CGCs were seeking to change or adopt a new FHx collection tool, 44.9% to change or adopt a new cancer screening tool, and 41.8% to change or adopt a pedigree drawing software tool (Table 2). Conversely, few genetic counselors were seeking to change or adopt health IT tools for patient communication (7.6%), gene test panel ordering (9.9%), and family communication (13.9%).

### 3.4 Projected future utilization of health IT among CGCs

Health IT utilization among CGCs in the future was calculated as a combination of CGCs currently using health IT with those actively seeking to adopt a new tool. These rates were higher compared to current utilization rates. Seven of 10 health IT tools are projected to be used by a majority (greater than 50%) of CGCs (Table 2). All (100.0%) CGCs were projected to use an EHR, followed by 91.1% of CGCs that are projected to use genetic risk assessment tools. Over three‐quarters of CGCs are projected to use FHx collection tools (78.9%) and pedigree drawing software (77.6%), respectively. Two thirds (66.4%) are projected to use telegentics software, and just over half are projected to use cancer screening tools (58.6%) and gene test panel ordering tools (52.0%). Only about one third are projected to use a patient communication tool (36.4%) or patient education tool (30.9%). Only 19.0% are projected to use a family communication tool.

## 4 DISCUSSION

Though many health IT resources are available to help CGCs provide cancer genetic counseling, little is known about their utilization collectively and individually. To address this gap, we conducted this study to gain insight into which health IT tools CGCs use, their satisfaction levels with these tools, and whether they are considering adopting or switching tools. Our findings can inform future health IT research and development.

We found that CGCs who use health IT tools are in the minority among their colleagues, with the exception of EHRs, risk assessment tools, and pedigree drawing software. A prior study by Zierhut *et. al*. found that 68.3% of genetic counselors used telemedicine, whereas we found that only 33.6% of CGCs currently use telegenetics (Zierhut, MacFarlane, Ahmed, & Davies, 2018). This discrepancy is possibly because the Zierhut definition of telemedicine included the use of telephones, whereas our definition is limited to video communication technologies. Similarly, Terry *et. al*. found that 62.7% self‐described telegenetics providers used live video conferencing (Terry et al., 2019). Our survey only assessed telegenetics (i.e., video) adoption limited to CGCs (as opposed to genetic counselors across other specialties).

Among participants in our survey, EHRs were the most widely used health IT product by CGCs, likely because most healthcare organizations that employ genetic counselors have also adopted EHRs. This is reflected by our finding that Epic is most commonly used among CGCs. While Epic has only a 28% overall market share in healthcare, it has the majority market of large healthcare organizations and academic medical centers, both of which are also most likely to hire clinical genetic counselors (KLAS Research, 2019; National Society of Genetic Counselors, 2018). Health IT tools to conduct risk assessment are also widely used among CGCs, likely because the risk models and calculations are complex and require the use of an electronic tool to complete effectively in clinic (Gail et al., 1989; Tyrer et al., 2004). Furthermore, there may be a lower barrier to adopt as many these risk models and calculators are freely available online or are included in pedigree drawing software the CGC is already using. Given that use of health IT among CGCs is projected to grow rapidly (Cohen et al., 2019; Insights Team, 2019), results about utilization will need to be updated in an ongoing manner to help track changes in health IT adoption over time.

With regards to satisfaction, gene test panel ordering tools had the highest satisfaction rate, possibly because these tools are provided for free by testing laboratories in order to make the test ordering process easier for CGCs (“Medical Genetic Testing Experts Trust Invitae” 2019; “Genetic Testing for Hereditary Cancer Ambry Genetics” 2019; “Awareness, Education and Support for Hereditary Cancer‐my Support360”). By providing a simple, satisfying order experience for the CGC, more CGCs may be willing to send genetic tests to their lab as opposed to a competing lab that has poor user experience. Thus, for testing labs there is likely a strong financial incentive to create a health IT solution with a positive user experience. Conversely, EHRs had the highest dissatisfaction rate among CGCs, likely the result of EHR vendors not designing their software for CGCs workflows (as opposed to genetic test ordering software) (Shoenbill, Fost, Tachinardi, & Mendonca, 2014). Given the scarcity of CGCs practicing in the United States (“Genetic Counselors: occupational Handbook: U.S. Bureau of Labor Statistics”, 2019), EHRs are likely often selected by the healthcare organization with little or no input from CGCs.

The survey revealed that nearly half of CGCs who responded to the survey were seeking to adopt or change their FHx collection tool, cancer screening tool, or their pedigree drawing software. This included nonusers seeking to adopt the technology, and current users seeking to change their current approach. Of note, even though there are several FHx collection tools commercially available to CGCs, a substantial lack of adoption still exists (Welch et al., 2018). This presents an opportunity for further research and development to better understand what CGCs are looking for in FHx collection tools and why the current technologies are not satisfying their needs in order to create more effective health IT solutions FHx collection tools for CGCs. This may include gaining a deeper understanding of CGCs’ specific “pain points” with existing health IT tools in order to determine what improvements can be made to better support delivery of cancer genetic counseling through health IT. More broadly, it would be necessary to consider additional factors outside of the tool characteristics that may influence the likelihood for a CGC to change or to adopt a health IT product such as cost, accessibility, time, or ability to influence a decision. Additional qualitative analysis could explore these insights in greater detail.

Furthermore, one potential opportunity to expand the use of health IT among CGCs is through traceback testing, which has been promoted as a way to improve the detection of families at risk for hereditary cancer (Samimi et al., 2017). CGCs often provide a letter for patients to send to at‐risk relatives to inform them of their potential risks. Despite health IT resources available to facilitate this process, few have taken advantage or are seeking to adopt such technology (Schmidlen, Schwartz, DiLoreto, Kirchner, & Sturm, 2019). Many CGCs indicated they were not aware that such health IT existed, highlighting the need for providing better education regarding health IT resources available to CGCs.

With regards to study limitations, only 128 CGCs responded to our survey questions. As a result of this limited sample, it is highly likely that some health IT tools used among CGCs were not captured. However, given that there are approximately 1,200 practicing cancer CGCs in the United States who meet our inclusion criteria, (“Genetic Counselors: Occupational Outlook Handbook:: U.S. Bureau of Labor Statistics”, 2019) our study sample likely represents approximately 10% of the entire cancer genetics provider population. We compared our demographic data with the National Society of Genetic Counselors (NSGC) Professional Status Survey 2018 to determine whether our sample provides a representative sample of CGCs (National Society of Genetic Counselors, 2018). Another limitation is that the 10 categories of health IT tools that we explored were selected internally by our team after reviewing literature and studying CGC workflows. A more systematic approach would have been to first conduct an assessment of all health IT used by CGCs and then to inductively form categories based on the data. Furthermore, we use a mixed‐mode approach, asking individuals to complete qualitative information about their quantitative response. The qualitative information is outside of the scope of the present study but may help contribute to better understanding of the frequency of use, the levels of satisfaction, and opportunities to improve tools. Finally, we measured satisfaction only from the perspective of the CGC. A more comprehensive view of tool satisfaction would also include feedback from patients, physicians, nurses, healthcare administrators, health IT managers, as well as other individuals that interact with the health IT tool.

## 5 CONCLUSION

Health IT has substantial room for expansion among CGCs. While EHRs and cancer risk model software are the most widely used solutions currently, health IT tools that facilitate FHx collection, cancer screening, pedigree management, and telegenetics software offer the greatest opportunities for research as well as health IT tool innovation and development. Additional research is needed to understand specific barriers and opportunities for these technologies, leading to the development of health IT products that best fit the needs of CGCs.

## CONFLICT OF INTEREST

Jordon Ritchie, Caitlin Allen, Michelle Nichols, Steven Lauzon, and Chanita Hughes Halbert have no conflicts of interest. Joshua Schiffman, Heath Morrison, and Brandon Welch are co‐founders and shareholders of ItRunsInMyFamily.com, Inc a web‐based family health history tool. Heath Morrison and Brandon Welch are co‐founders and shareholders of Dokbot, LLC, a chatbot for data capture. Brandon Welch is the founder and shareholder of Doxy.me, LLC a telemedicine platform.

## AUTHOR CONTRIBUTIONS

Jordon Ritchie and Brandon Welch contributed to the conception and design of the work. Jordon Ritchie, Caitlin Allen, Michelle Nichols, Steven Lauzon, and Brandon Welch contributed to the acquisition, analysis, or interpretation of data for the work. Jordon Ritchie, Caitlin Allen, Michelle Nichols, Heath Morrison, Steven Lauzon, Joshua Schiffman, and Chanita Hughes Halbert Brandon Welch contributed to drafting the work or revising it critically for important intellectual content and final approval of the version to be published.

## Data Availability

The data that support the findings of this study are available from the corresponding author upon reasonable request.
